# Physiopathology of fibrinolysis in sepsis-induced disseminated intravascular coagulation: Emerging mechanisms and pharmacological targets

**DOI:** 10.1016/j.aicoj.2025.100008

**Published:** 2026-01-16

**Authors:** Marine Tschirhart, Maeva Martin, Anaïs Curtiaud, Eduardo Angles-Cano, Ferhat Meziani, Florence Toti, Julie Helms

**Affiliations:** a INSERM (French National Institute of Health and Medical Research), UMR 1260, Regenerative Nanomedicine (RNM), FMTS, Strasbourg, France; b Université de Strasbourg (UNISTRA), Faculté de Médecine, Fédération Hospitalo-Universitaire (FHU) TARGET, Hôpitaux Universitaires de Strasbourg, Service de Médecine Intensive-Réanimation, Nouvel Hôpital Civil, Strasbourg, France; c Université Paris Cité - INSERM U-1140, Innovative Therapies in Haemostasis, 75006 Paris, France

**Keywords:** Disseminated intravascular coagulation, Fibrinolysis, Coagulopathy, Sepsis

## Abstract

Septic shock represents the most severe form of an infection, marked by a dysregulated host response that can lead to multiple organ failure and death. Among these patients, 30–40% develop disseminated intravascular coagulation (DIC), a life-threatening complication associated with a 60% increase in mortality. DIC is characterized by widespread activation of the coagulation cascade, resulting in disseminated microthrombi and a hypercoagulable state. This prothrombotic profile arises from the upregulated expression of tissue factor by endothelial cells, monocytes, and neutrophils, combined with insufficient regulation by endogenous anticoagulant pathways. In addition, a profound impairment of fibrinolysis further contributes to this imbalance. Initially, this fibrinolytic insufficiency was attributed to elevated plasma levels of plasminogen activator inhibitor-1 and thrombin-activatable fibrinolysis inhibitor. More recently, it has been shown that DIC-associated fibrinolytic insufficiency during septic shock involves plasminogen degradation driven by neutrophil elastase carried by neutrophil extracellular traps circulating in patients’ plasma.

The failure to resolve this hypercoagulable state and restore hemostatic balance has emerged as a key determinant of poor outcomes in DIC. Therefore, elucidating the mechanisms underlying fibrinolytic insufficiency is very important both to identify at-risk patients and to treat DIC.

This review provides an overview of the most recent advances in our understanding of fibrinolytic dysregulation in sepsis-induced DIC, with a particular focus on emerging molecular mechanisms and their implications for the identification of novel pharmacological targets.

## Introduction

During sepsis, the host response to pathogen invasion involves the activation of several defense mechanisms. It is now well established that inflammation invariably leads to the activation of coagulation, and that hypercoagulation and thrombosis are associated with inflammatory signaling [1]. The interaction between immunity and hemostasis, known as “immunothrombosis”, enables pathogen recognition, containment, and destruction, and was initially described as a biological defense mechanism of the host against infection [2].

However, in some cases, these defense mechanisms become dysregulated, triggering uncontrolled inflammation that leads to extensive tissue damage and excessive activation of the coagulation cascade. Intense activation of vascular cells—particularly leukocytes, platelets, and endothelial cells—leads to endothelial dysfunction, rendering the endothelium pro-adhesive, pro-thrombotic, and anti-fibrinolytic. This is further amplified by a marked increase in circulating mediators that sustain feed-forward, intertwined inflammatory and coagulation loops [3]. Excessive coagulation is further sustained by defective anticoagulant systems and fibrinolytic insufficiency, ultimately leading to disseminated intravascular coagulation (DIC) in 30–40% of cases of sepsis and septic shock [4].

In 2001 [5], and updated on 2025 [6], DIC was defined by the International Society of Thrombosis and Hemostasis (ISTH) as “an acquired life-threatening intravascular disorder characterized by systemic coagulation activation, dysregulated fibrinolysis, and endothelial injury, resulting in microthrombosis”. In sepsis, DIC plays a key role in amplifying the prothrombotic state and promoting the development of multiple organ failure, and is associated with markedly increased mortality, with rates ranging from 31% to 86%, and consistently exceeding those observed in septic patients without DIC [7,8]. Sepsis-induced coagulopathy (SIC) represents an early and fast-evolving phase of coagulation activation, preceding overt DIC. It was proposed by the ISTH to identify patients with sepsis-related coagulation disorders at an earlier, potentially reversible stage [9]. The SIC score combines platelet count, prothrombin time (expressed as INR), and the Sequential Organ Failure Assessment (SOFA) score. A positive SIC score reflects the onset of thrombin generation and microvascular thrombosis, but without the full consumptive pattern seen in DIC. Clinically, SIC identifies patients with increased risk of organ dysfunction and mortality, and who might benefit from early therapeutic interventions. However, the fibrinolytic status of patients with SIC has never been specifically characterized, despite its potential importance in the dissemination of a local and controlled defense mechanism of coagulation into systemic circulation favoring coagulopathy.

The clinical manifestations of DIC are highly heterogeneous, ranging from thrombotic complications to bleeding events. However, sepsis-induced DIC is predominantly characterized by a prothrombotic profile, which can manifest as peripheral ischemia, including necrosis of the fingertips and toes [10,11]. Additionally, microthrombosis contributes to organ dysfunction by impairing visceral perfusion.

This review provides an overview of recent advances in our understanding of the dysregulated fibrinolysis observed in sepsis-induced DIC. It highlights emerging mechanistic insights—from neutrophil-mediated plasminogen degradation to antifibrinolytic mediator imbalance—and explores how these discoveries may inform the development of new pharmacological strategies to restore hemostatic balance and improve outcomes.

## The hypercoagulable response to pathogens

**Fig. 1:**
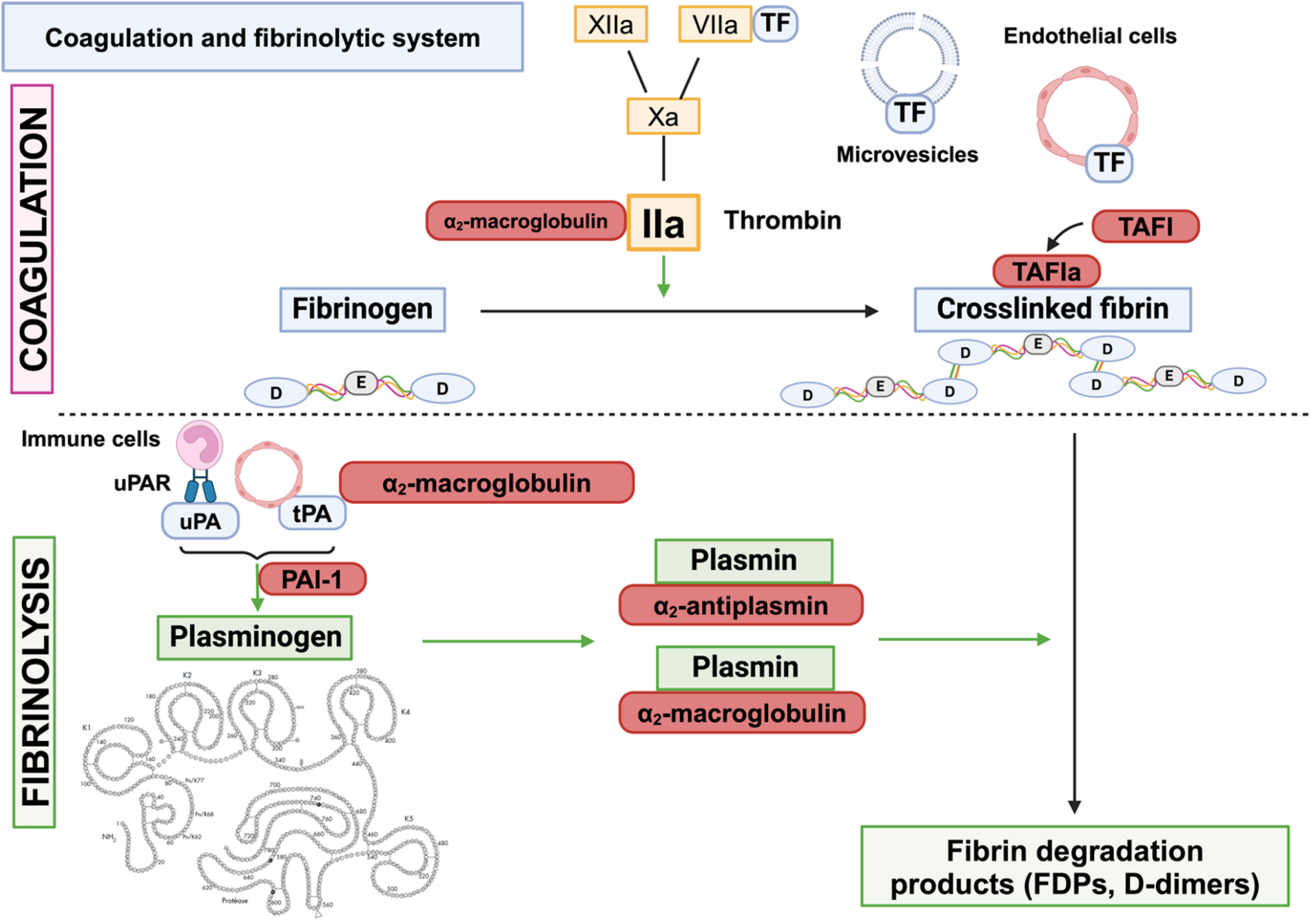
The coagulation and fibrinolytic system. FDPs: fibrin degradation products; PAI-1: plasminogen activator inhibitor-1; TAFI: thrombin-activatable fibrinolysis inhibitor; TF: tissue factor; tPA: tissue plasminogen activator; uPA: urokinase plasminogen activator

Host recognition of the pathogen leads to intense production of pro-inflammatory cytokines, which target the endothelium, neutrophils and monocytes. Indeed, pro-inflammatory cytokines induce the transcription and membrane expression of tissue factor (TF), the cellular initiator of the coagulation cascade (Fig. 1). TF, expressed by endothelial cells, platelets, monocytes, and lymphocytes, is the receptor for circulating coagulation FVII/VIIa [12]. At the cell surface, the binding of FVIIa to TF initiates the sequential formation of blood coagulation complexes.

The formation of these complexes relies on the vitamin K-dependent post-translational modification of γ-carboxyglutamic (GLA) residues as well as the correct folding and activity of coagulation factors (FVII, FIX, FX, Prothrombin) and activated protein C (APC). The GLA domain of these factors binds to phosphatidylserine exposed by the plasma membrane of activated cells in a Ca^2+^-dependant manner and participate in the process of coagulation and anticoagulation by APC. On the phosphatidylserine surface of activated platelets, the TF-FVIIa complex activates FIX to form the tenase TF-FVIIa-IXa complex which activates FX thus forming the FIXa-VIIIa-Xa complex with factor VIIIa as a cofactor. FXa and FVa form a prothrombinase complex, which allows FII (prothrombin) to be converted to FIIa resulting in massive production of thrombin. Thrombin then cleaves plasma fibrinogen into fibrin monomers, which are subsequently transformed into an insoluble fibrin network by FXIIIa [13].

Initially, the excessive activation of coagulation is a central driver of DIC. However, this procoagulant state persists over time due to the progressive failure of the endogenous anticoagulant system. Plasma levels of natural anticoagulants—including protein C, thrombomodulin, antithrombin, and tissue factor pathway inhibitor—decline markedly, primarily due to their consumption in response to overwhelming coagulation activation and, in some cases, reduced synthesis or degradation by inflammatory proteases.

Additional mechanisms further amplify the procoagulant response at sites of infection. Activated neutrophils release both microvesicles (MVs) and neutrophil extracellular traps (NETs), which promote clot formation.

MVs are submicron vesicles released in blood from the plasma membrane of activated cells. MVs expose phosphatidylserine, and, in some cases, TF, when it is expressed by their parental cell. MVs constitute an additional catalytic surface for the formation of the coagulation complexes and behave as cellular effectors prompting the expression of TF in autocrine and paracrine loops.

NET release—known as NETosis—is a late-stage neutrophil response aimed at trapping and disabling pathogens. These web-like structures are made of decondensed DNA coated with citrullinated histones and enzymes—such as myeloperoxidase and neutrophil elastase— and contribute to both antimicrobial defense and local thrombosis [14,15]. The activation of the contact phase by the decondensed DNA fibers of NETs represents another procoagulant pathway, eventually amplified by MVs, which can bind to these fibers. NETs also amplify the hypercoagulation by degrading anticoagulant proteins. Finally, histones borne by NETs induce thrombin generation in a platelet-dependent manner and favor the proteolysis of TFPI, both key in the exaggerated procoagulant response [16].

In DIC, sustained hypercoagulability is driven in part by impaired fibrinolysis, which fails to counterbalance the initial procoagulant response. Consequently, fibrin accumulates and persists within the microvasculature, contributing to organ dysfunction and worsening the overall prognosis. This persistence highlights the pivotal role of fibrinolytic insufficiency in the pathophysiology of DIC. To fully appreciate the impact of this imbalance, it is essential to first understand the normal functioning of the fibrinolytic system under physiological conditions.

## The fibrinolytic response in healthy individuals

The fibrinolytic system plays a crucial role in dissolving microthrombi and maintaining vascular permeability [17] (Fig. 1). At the core of this process is the conversion of plasminogen into plasmin—a proteolytic enzyme—on the surface of fibrin and cell membranes. This activation is mediated by plasminogen activators and tightly regulated by specific protease inhibitors to ensure a balanced response.

Native plasminogen, also known as Glu-plasminogen, is a single-chain glycoprotein composed of 791 amino acids. In plasma, it circulates in free form or bound to α2-antiplasmin, histidine-rich glycoprotein, or fibrinogen. Structurally, plasminogen consists of seven domains: a pre-activation peptide, five kringle domains (K1–K5), and a serine protease domain responsible for its enzymatic activity.

Plasminogen is converted into plasmin by two key activators: tissue-type plasminogen activator (tPA), mainly secreted by activated endothelial cells, and urokinase-type plasminogen activator (uPA), produced by immune cells, epithelial cells, megakaryocytes, and other tissues [17]. On the surface of fibrin, plasminogen and tPA bind through specific interactions—the lysine-binding sites on kringle domains K1 and K4 of plasminogen, and the finger domain of tPA—forming a ternary complex that promotes plasmin generation [18].

At the cell surface, uPA binds to its receptor uPAR, while plasminogen interacts with lysine residues on various membrane-associated glycoproteins. Notably, only trace amounts of tPA and uPA circulate in their free forms; in plasma, they are predominantly found in complex with their primary inhibitor, plasminogen activator inhibitor-1 (PAI-1). Free, active tPA and uPA are typically confined to sites of fibrin deposition or inflammation, where they locally initiate fibrinolysis.

### Acceleration and amplification of fibrinolysis

Once formed at the site of fibrin deposition, plasmin accelerates and amplifies fibrinolysis by exposing new plasminogen-binding sites—specifically carboxy-terminal lysine residues (C-terminal Lys). When plasminogen binds to these sites, it undergoes a conformational change to its open form, which allows more efficient activation by nearby tissue plasminogen activator (tPA). This localized increase in plasminogen binding initiates a cascade in which enhanced plasmin generation further amplifies fibrin degradation and promotes efficient clot lysis [19,20]. This positive feedback loop—driven by the multiplication of C-terminal lysines and the enhanced binding of open-conformation plasminogen—is a key mechanism behind the rapid amplification of fibrinolysis. At the cellular level, a second accelerating mechanism occurs when leukocyte-derived single-chain urokinase (sc-uPA) binds to its receptor, uPAR [19]. The first traces of plasmin convert sc-uPA into its active two-chain form, further promoting local plasmin generation. In parallel, components of the intrinsic coagulation pathway, such as activated FXII and kallikrein, support fibrinolysis indirectly by stimulating bradykinin production [21,22]. Bradykinin is a potent inducer of endothelial tPA release, linking coagulation, inflammation, and fibrinolysis.

Interestingly, under inflammatory conditions, MVs bearing uPA can promote localized plasmin generation at plasminogen-rich surfaces, such as fibrin deposits, the extracellular matrix, and activated platelets. This MV-driven fibrinolytic crosstalk concentrates plasmin activity at thrombogenic sites, enhancing the efficiency of fibrin clearance while potentially offsetting the procoagulant effects of MVs themselves [23,24]. In this context, plasminogen activation on cellular and fibrin surfaces depends on heterotypic interactions between target cells and MVs displaying the uPA/uPAR complex [25]. Notably, the uPA linked to uPAR on MVs is shielded from inhibition by PAI-1, making these vesicles highly effective plasminogen activators—even at distant sites of inflammation or fibrin accumulation [26]. This mechanism represents a promising therapeutic target, enabling localized fibrinolysis while minimizing the risk of systemic activation.

The involvement of multiple targets within the fibrinolytic system has been validated in genetically engineered mice, underscoring their relevance in regulating thrombus formation and resolution. tPA deficiency impairs blood clot lysis, while uPA knockdown leads to sporadic fibrin deposition. Mice lacking both tPA and uPA exhibit significantly increased fibrin accumulation compared to wild-type controls, with detrimental effects on growth, fertility, and survival [27]. Notably, tPA-deficient mice display an impaired immune response, resulting in higher bacterial loads, increased systemic dissemination, and reduced survival [28].

### Attenuation of the fibrinolytic response

The fibrinolytic system is tightly controlled by finely tuned regulatory pathways involving the inhibition of plasminogen activation, the neutralization of plasmin activity, and the modulation of fibrin network structure. Two key serine protease inhibitors (serpins), PAI-1 and α2-antiplasmin, play central roles in this regulation. PAI-1 inhibits plasminogen activation by targeting free tPA and uPA, thereby limiting plasmin generation. In contrast, α2-antiplasmin acts as a direct inhibitor of plasmin and can be covalently cross-linked to fibrin via its C-terminal region, a process that enhances its ability to localize inhibition and prevent thrombus expansion [29].

In cases of overproduction of tPA or plasmin, other major plasma inhibitors, such as α2-macroglobulin, may also contribute to the regulation of fibrinolysis [30]. Additionally, it has been suggested that thrombin-activatable fibrinolysis inhibitor (TAFI), a plasma carboxypeptidase, synthetized by the liver that circulates in plasma, has been proposed as another important regulator. Once activated by thrombin or plasmin, TAFIa—an exopeptidase—removes C-terminal lysine residues from proteins. In this context, its primary substrate is likely to be degraded fibrin, where cleavage of terminal lysine reduces plasminogen binding and limits further plasmin generation at the clot surface [31]. However, the relevance of this mechanism to thrombosis remains uncertain, as findings across experimental and clinical studies have been inconsistent [[32], [33], [34], [35]]. Rather than playing a central role in fibrinolysis control, TAFI may exert more significant effects through its anti-inflammatory properties [36].

PAI-1 is the main inhibitor of the initial phase of fibrinolysis, acting rapidly on both t-PA and uPA to form a 1:1 complex. PAI-1 is mainly produced by endothelial cells and megakaryocytes. PAI-1 is mainly stored in platelets [37], though it can also be secreted to blood flow or deposited on the subendothelial matrix. The active form of PAI-1 is stabilized in vivo by the association with vitronectin in plasma and with the extracellular matrix [38]. Beyond the control of fibrinolysis, PAI-1 exerts a protective role in immune responses. In PAI-1 deficient mice challenged by LPS or staphylococcal enterotoxin B an increased production of IFN-γ was observed [39]. It has also been demonstrated that PAI-1 improves the host’s defense against a Klebsiella pneumonia bacterial infection [40]. In addition, PAI-1 deficient mice showed increased bacterial loads in distant sites of infection and increased organ failures [41]. Altogether, these data suggest that the anti-fibrinolytic activity of PAI-1 impacts the initial site of infection and likely contributes to limiting bacterial dissemination through fibrin clot entrapment.

The α2-antiplasmin is a circulating glycoprotein synthesized in the liver and a direct inhibitor of plasmin at the C-terminal lysine residues. Its binding to plasmin constitutes an irreversible complex known as plasmin-antiplasmin (PAP), which neutralizes plasmin’s fibrinolytic activity and regulates the later stages of fibrinolysis [20,42]. The presence of the PAP complex in the circulation represents a direct indication of plasmin production in vivo.

Furthermore, α2-antiplasmin can be cross-linked to fibrin by factor XIIIa, thus preventing plasmin from binding to fibrin and stabilizing the fibrin network [43].

The α2-macroglobulin is a glycoprotein primarily produced in the liver, but also by other cell types such as fibroblasts and macrophages. By inhibiting plasmin, it acts as a substitute for α2-antiplasmin fibrinolysis but also contributes to hemostasis by inhibiting thrombin [30]. By binding to plasmin, it forms a complex enabling its elimination by phagocytosis. Additionally, it has been demonstrated that α2-macroglobulin can inhibit t-PA, urokinase, and kallikrein, which has a significant impact on fibrinolysis [30,44].

In summary, PAI-1, α2-antiplasmin, and α2-macroglobulin are key regulators of fibrinolysis, acting at different stages of the process. PAI-1 functions upstream by inhibiting plasminogen activators and thereby preventing the conversion of plasminogen into plasmin. α2-antiplasmin and α2-macroglobulin act both during ongoing plasmin generation—by directly inhibiting plasmin—and downstream by targeting the fibrin network, limiting the local binding and activity of profibrinolytic factors. Together, these three inhibitors ensure the precise spatial and temporal control of fibrinolytic activity.

The complex interplay between coagulation and fibrinolysis ensures a balance between clot formation and dissolution to avoid the formation of microthrombi. However, in pathological situations such as DIC in septic shock, this balance is disrupted. The associated fibrinolytic insufficiency blunts the control of the early pro-coagulant response and later exaggerated coagulation leading to multiple organ failure.

## Fibrinolytic insufficiency in sepsis-induced DIC: an evolutive concept

**Fig. 2:**
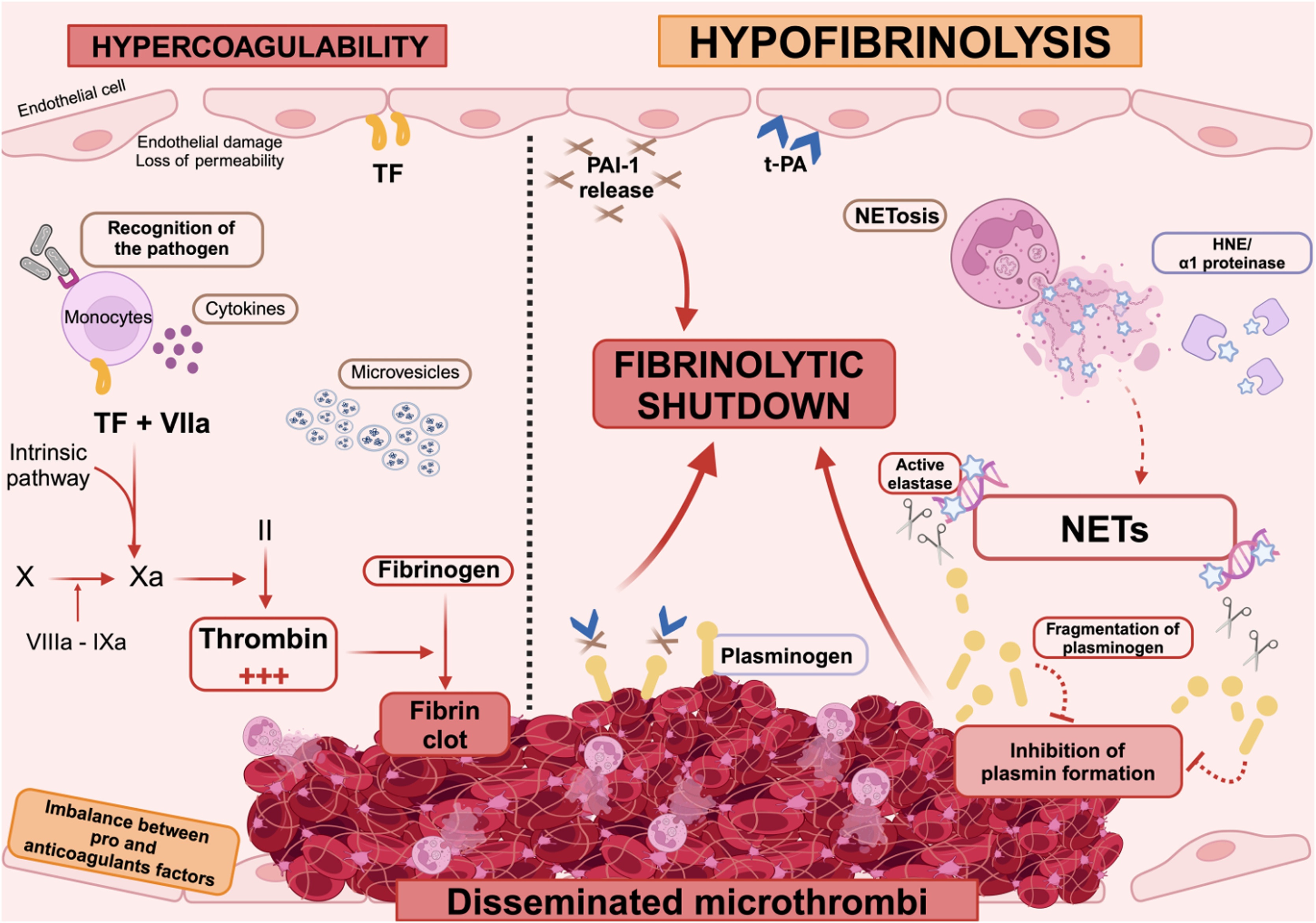
Mechanisms of fibrinolytic insufficiency in sepsis-induced disseminated intravascular coagulation. Pathogen recognition by the innate immune system triggers a massive release of pro-inflammatory cytokines and activation of vascular cells, ultimately leading to endothelial dysfunction. This excessive activation induces a hypercoagulable state, characterized by excessive thrombin generation and impairment of natural anticoagulant pathways, thereby promoting the formation of widespread microthrombi. This hemostatic imbalance is further exacerbated by impaired fibrinolysis. Indeed, the physiological activation of plasminogen into plasmin by tPA is inhibited due to the overexpression of PAI-1. In addition, abnormal plasminogen fragments have been identified in the plasma of septic patients compared to healthy individuals. This degradation is driven by neutrophil elastase bound to circulating NETs, which escapes inhibition by α1-proteinase inhibitor. This pathological cleavage reduces the availability of functional plasminogen, impairs its conversion into plasmin, and thereby contributes to the fibrinolytic insufficiency observed in septic DIC. HNE: human neutrophil elastase; NETs: neutrophil extracellular traps; PAI-1: plasminogen activator inhibitor-1; TF: tissue factor; tPA: tissue plasminogen activator.

In DIC, the tightly intertwined mechanisms regulating fibrinolysis become dysregulated, resulting in a marked reduction—or even complete insufficiency—of fibrinolytic activity. This imbalance involves both impaired activation and excessive inhibition of fibrinolytic and procoagulant factors and is closely linked to aberrant inflammatory responses (Fig. 2). This convergence of coagulation and inflammation is characteristic of immunothrombosis [45].

### The role of PAI-1 in fibrinolytic insufficiency in septic DIC

DIC-associated fibrinolytic insufficiency was initially linked to elevated circulating levels of PAI-1, released in response to thrombin stimulation, which triggers PAI-1 secretion from platelet and endothelial cell granules [37]. This early rise in PAI-1 is accompanied by transient increases in plasma concentrations of tPA and PAP complexes, indicating an initial activation of fibrinolysis. However, as PAI-1 levels continue to rise, tPA concentrations subsequently decline, leading to reduced plasminogen activation and a net antifibrinolytic effect. This biphasic response has been well documented in experimental models of endotoxemia in both chimpanzees and humans [46,47]. Several studies have underscored the pivotal role of the systemic inflammatory response in regulating fibrinolysis, particularly through the release of PAI-1 and tPA by endothelial cells activated in response to lipopolysaccharide (LPS) [48]. For instance, endotoxin exposure in healthy subjects has been shown to induce an early rise in plasma TNF-α and IL-6 levels, preceding the increase in tPA and PAP complexes, which is subsequently counterbalanced by a delayed but sustained elevation in PAI-1 concentrations [46,49]. In LPS-treated primates, initial increases in tPA are followed by a plateau of PAI-1 levels lasting approximately two hours before a sharp surge is observed [47].

Notably, administration of anti-TNF-α—but not anti-IL-6—antibodies significantly attenuated the increase in both tPA and PAI-1, strongly implicating TNF-α as a key mediator of the fibrinolytic response during endotoxemia. In addition, TNF-α has been shown to enhance uPA expression in endothelial cells, suggesting a possible role in pericellular proteolysis. In murine models, intraperitoneal injection of LPS or TNF-α leads to a rapid and pronounced induction of PAI-1 transcription across multiple organs—including the aorta, kidneys, lungs, and liver—highlighting the importance of restoring PAI-1 stores and the need for tightly regulated PAI-1 expression during sepsis [50].

In sepsis-induced DIC, several trials support a key role of PAI-1 plasma elevation on patients’ outcome [[51], [52], [53], [54], [55], [56]]. In a prospective monocentric study, a significant increase in PAI-1 expression was observed in patients with severe versus moderate sepsis, which correlated with increased mortality [57]. Similarly, a case-control study showed a correlation between decreased fibrinolytic activity in patients with DIC and increased mortality [58]. While PAI-1 is now widely recognized as the primary mediator in fibrinolysis inhibition during DIC, other anti-fibrinolytic pathways or neutrophil elastase may also contribute.

### TAFI and fibrinolytic insufficiency in septic DIC: current insights and uncertainties

TAFI regulates fibrinolysis by removing C-terminal lysines from fibrin, thereby limiting plasmin generation and stabilizing clots. While physiologically relevant, its role in the fibrinolytic insufficiency observed in sepsis-induced DIC remains unclear. Clinical and experimental studies have yielded inconsistent results—some reporting reduced TAFI levels associated with increased fibrinolytic activity, others finding no significant differences depending on DIC status or organ failure severity [33,34]. These discrepancies may reflect variations in measurement methods, sampling timing, animal models, or patient stratification.

In rodents, TAFI inhibition has led to both increased and decreased fibrinolytic activity depending on the species, further complicating interpretation [35,59]. Overall, current evidence does not support a consistent contribution of TAFI to fibrinolytic insufficiency in septic DIC. Instead, TAFI may play a more prominent role in modulating inflammation, particularly through its regulation by thrombin, thrombomodulin, and neutrophil elastase [60]. Its precise involvement in fibrinolytic insufficiency remains uncertain and appears less central than initially suspected. This highlights the need to refocus on other mechanisms contributing to hypofibrinolysis in septic DIC, particularly those supported by more consistent clinical and experimental evidence.

### Recent advances in sepsis-induced DIC hypofibrinolysis

In patients with septic shock, the drop in circulating plasminogen may result in part from consumption by fibrinolysis secondary to DIC, as evidenced by the increase in D-dimers. However, the persistence of microthrombi in DIC also indicates a failure of the fibrinolytic system. Previous reports strongly suggest that plasminogen binding to cell or fibrin surfaces is the rate limiting factor for fibrinolysis and that a circulating plasminogen concentration below 1 µmol/L can lead to insufficient plasminogen binding and fibrinolysis deficiency [[61], [62], [102]. In healthy individuals, plasminogen concentrations typically range from 1.5 to 2 µmol/L (138–184 µg/mL), providing sufficient substrate for tPA-mediated plasmin generation and effective fibrin degradation. Yet, the impact of NETs on fibrinolysis has only recently come into focus, revealing another macromolecular interface at the crossroads of immunothrombosis. Initial evidence came from the observation that NETs integrate into the fibrin matrix, reducing the surface accessibility for plasminogen and plasmin binding [63]. A pivotal clinical study further highlighted this interference: in patients with sepsis-induced DIC, plasma contained abnormal plasminogen fragments not found in healthy individuals [64]. This atypical proteolytic cleavage was driven by neutrophil elastase bound to circulating NETs fragments, which shielded the enzyme from its natural inhibitor, α1-proteinase inhibitor. Consequently, full-length plasminogen was degraded, limiting its availability for activation into plasmin and thereby impairing fibrinolysis. Together, these findings support a novel mechanism of plasminogen deficiency driven by NET-associated elastase activity—one that may significantly contribute to the hypofibrinolytic state in septic coagulopathy.

Fibrinolytic insufficiency plays a crucial role in the complex evolution of DIC. While coagulation is excessively activated, fibrinolysis, the natural process of clot dissolution is inhibited. This inhibition favors the formation and spread of intravascular microthrombi. The imbalance between fibrin formation and dissolution directly contributes to organ failure. Fibrinolytic insufficiency that recently appeared crucial in the evolution of DIC, represents a significant therapeutic target for improving clinical outcomes and limit DIC-associated organ failure. Understanding of the mechanisms behind fibrinolytic insufficiency could lead to improved patients’ stratification, monitoring, and the proposition of new therapeutic strategies aimed at restoring the balance between coagulation and fibrinolysis.

## Diagnosis and monitoring of the fibrinolytic insufficiency: emerging actors

The diagnosis scores established by the International Society on Thrombosis and Haemostasias (SIC and ISTH scores) and the Japanese Association for Acute Medicine (JAAM) are widely recognized for the diagnosis of DIC in sepsis and septic shock [65,66]. Such scores however fail to detect the “pre-DIC” stage of asymptomatic and clinically undetectable DIC, where coagulation remains compensated by natural anticoagulants. Despite the absence of overt clinical signs, pre-DIC patients exhibit disease severity and mortality rates comparable to those with overt DIC at the time of ICU admission [8].

To improve early diagnosis, novel biomarkers have been proposed [67]. Among them, procoagulant MVs of endothelial and leukocyte origin indicate early cellular activation and are significantly elevated in the initial phases of septic DIC, preceding the positivity of conventional scores [8,68]. Additionally, during the NETosis process, neutrophil fluorescence (NEUT-SFL) has been identified as a reliable marker of neutrophil activation, also associated with DIC [69]. Yet the impact of NETosis on fibrinolysis is not fully deciphered.

In the time course of DIC, the monitoring of fibrinolysis-related biomarkers, including PAI-1, α2-antiplasmin, TAFI and D-dimers, have gained interest. Elevated PAI-1 plasma concentration correlates with increased mortality in DIC and may serve as both diagnostic and prognostic indicators [70,71]. While D-dimers have been proposed to evaluate the disease severity in pre-DIC and DIC patients, their specificity for DIC diagnosis can be altered by others physio pathological conditions such as age and sex [72]. Fibrin degradation products were reported more sensitive in the early DIC and to correlate with disease severity [73]. In advanced DIC, a decrease in α2-antiplasmin may reflect its consumption in response to hypercoagulation [58]. Moreover, PAP complexes appear stable and reliable markers of ongoing fibrinolytic activity of possible value for monitoring while their plasma concentration may vary with other underlying pathologies, such as diabetes and dyslipidemia explaining values discrepancies between trials [74]. In addition, TAFI concentration and carboxypeptidase activity were reported to drop drastically in pre-DIC and DIC patients, suggesting hypofibrinolysis, as confirmed in canine models of sepsis or after severe injury [33,75,76].

Beyond biomarker-based approaches, viscoelastic assays such as thromboelastography (TEG) and rotational thromboelastometry (ROTEM) offer an integrated whole-blood, point-of-care evaluation of clotting. By tracking real-time changes in blood viscoelasticity, they enable assessment of clot initiation, propagation, firmness, and dissolution, thereby providing an overview of both coagulation and fibrinolytic functions [77]. Fibrinolysis is quantified by a decrease in clot amplitude, after maximum clot firmness (ROTEM) or maximum amplitude (TEG) is reached [78]. Other ROTEM-derived parameters, such as the time from maximum velocity or maximum clot formation, were proposed as promising markers to predict mortality and disease severity in sepsis [79].

However, in the context of septic induced-DIC, fibrinolytic deficiency is far more common than hyperfibrinolysis, making the detection and analysis of low fibrinolytic activity by standard viscoelastic tests challenging. This stems from both the conditions of strong tissue factor activation used in these assays and their reliance on endogenous tPA, which drives sustained clot firmness, even when fibrinolysis is inhibited [77]. While viscoelastic assays are widely used in clinical practice to rapidly assess overall coagulation and fibrinolysis balances and guide transfusion, their low sensitivity to early or moderate alterations in fibrinolysis impairs a graduated assessment. Incorporating exogenous tPA or uPA have been developed to enhance the sensitivity of viscoelastic tests to fibrinolytic resistance. Yet, considerable interindividual variability in fibrinolytic potential among septic patients was reported [78]. Given the current limitations, assessment of complementary fibrinolysis-related biomarkers appears essential for timely and accurate diagnosis of septic DIC.

Unfortunately, currently available point-of-care (POC) devices Other than viscoelastic assays, have provides only fragmented information on the initiation and resolution of coagulation and fibrinolysis. POC tests for PT/INR (i-STAT / CoaguChek, Hemochron), platelet function analyzers (PFA-100 / Multiplate), or D-dimers lack both sensitivity and specificity for diagnosing septic DIC and are not validated for bedside use in this setting. Moreover, no single hemostatic parameter is currently able to diagnose DIC, which further limits the clinical utility of these point-of-care tests.

Therefore, despite numerous observations from both preclinical and clinical studies, no consensus has yet emerged for the early detection of DIC, which continues to hinder the development of effective therapies targeting fibrinolytic insufficiency. Yet, timely identification of septic DIC remains essential to allow appropriate patient stratification and to optimize the therapeutic window for interventions aimed at either curbing excessive coagulation or restoring impaired fibrinolysis.

## Current treatments and therapeutic perspectives

**Fig. 3:**
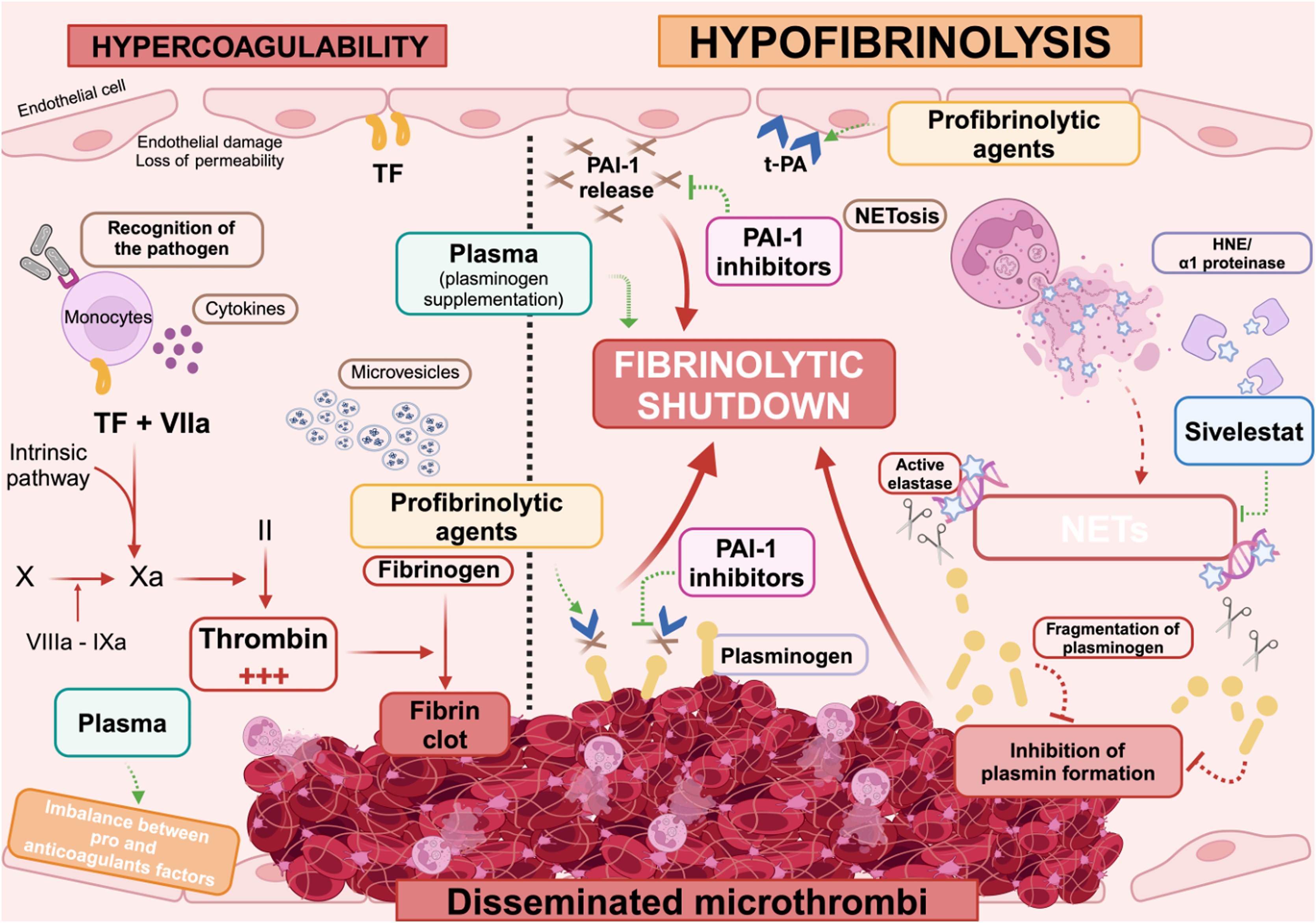
Targeting fibrinolytic insufficiency: potential therapeutic strategies in sepsis-induced disseminated intravascular coagulation. HNE: human neutrophil elastase; NETs: neutrophil extracellular traps; PAI-1: plasminogen activator inhibitor-1; TF: tissue factor; tPA: tissue plasminogen activator.

As with its diagnosis, a standardized treatment approach for fibrinolytic insufficiency in septic DIC has yet to be defined. The current management of DIC relies on three main pillars: (i) accurate and timely diagnosis, (ii) appropriate treatment of the underlying cause (sepsis), and (iii) control of excessive coagulation alongside restoration of fibrinolytic balance [10] (Fig. 3).

Given the predominantly prothrombotic nature of sepsis-induced DIC, several anticoagulant therapies have been evaluated with the goal of reducing microthrombi formation and improving organ perfusion. However, clinical trials have so far failed to demonstrate clear benefit, with anticoagulant treatments often associated with an increased risk of bleeding complications. For instance, recombinant activated protein C (Drotecogin alfa, Xigris®) initially showed promising results in the PROWESS trial [80] with reduced 28-day mortality and decreased D-dimer and interleukin levels, suggesting anticoagulant and immunomodulatory effects. Nonetheless, subsequent studies, including the PROWESS-SHOCK trial [81], failed to confirm any mortality benefit, while highlighting persistent hemorrhagic risk, ultimately leading to its market withdrawal in 2011. However, in both PROWESS and PROWESS-SHOCK trials, DIC was not a predefined inclusion criterion, and only *post hoc* analyses identified that approximately 20%–25% of patients in PROWESS and 15%–20% in PROWESS-SHOCK fulfilled the ISTH or JAAM criteria for overt DIC. The therapeutic effect of recombinant activated protein C was mainly observed in patients with marked coagulopathy in PROWESS but could not be confirmed in PROWESS-SHOCK, where most patients did not have overt DIC.

These findings emphasize that, when targeting DIC as a therapeutic objective, it is essential to select patients with biologically confirmed DIC rather than treating unselected populations with sepsis or septic shock. Such an approach was adopted in the SCARLET trial [82], which evaluated recombinant thrombomodulin in patients with sepsis-associated coagulopathy, ultimately failed to demonstrate a significant survival benefit. These observations highlight the importance of better characterizing the causes of the underlying coagulopathy, particularly the hypofibrinolytic state observed in septic DIC, as restoring fibrinolytic balance may represent a more effective and safer therapeutic target.

### Restoring the coagulo-fibrinolytic balance

Replacing or supplying plasma proteins in patients with low levels of coagulation factors and antithrombin to decrease bleeding risk and enhance heparin efficacy has been investigated. In a clinical trial enrolling 80 children with sepsis or septic shock in the non-overt DIC stage, early use of a combination of fresh-frozen plasma, low-dose heparin, and tranexamic acid improved survival and prevented the progression to overt DIC without increasing the bleeding risk [83]. Additionally, two comparative studies conducted on 31 and 112 patients with septic shock with or without DIC showed that an early plasma exchange reduced the imbalance between pro- and anticoagulant factors leading to septic coagulopathy [84,85].

### Increasing fibrinolytic function: PAI-1 inhibition and tPA antibodies intervention

Therapeutic agents targeting PAI-1 have attracted considerable interest, based on the rationale that inhibiting PAI-1 could enhance plasmin generation, promote fibrin degradation, and potentially prevent organ failure - as suggested by preclinical studies in models of arterial thrombosis. For instance, a monoclonal anti-PAI-1 antibody significantly reduced thrombus growth in rabbits [86], while a polyclonal PAI-1-inhibiting antibody showed a moderate reduction in thrombus size and partially restored blood flow in rats [87].

Although pioneering, these studies presented several limitations. First, they were conducted in models of macrovascular arterial thrombosis, which only partially reflect the microvascular thrombosis condition of DIC. Second, antibodies present low bioavailability [88]. Small-molecule PAI-1 inhibitors, such as Tiplatin, have shown antithrombotic efficacy in murine carotid and vena cava models, as well as in canine coronary arteries [[88], [89], [90]]. In a murine model of sepsis, Embelin, was associated with enhanced fibrinolysis and reduced pulmonary microthrombi formation [91]. However, the efficacy of PAI-1 inhibitors in restoring fibrinolysis in human sepsis-associated DIC remains to be demonstrated.

Other pro-fibrinolytic agents, such as uPA, have also shown promise. In a porcine model of E. coli–induced DIC, early administration of uPA restored blood flow and reversed organ damage [92]. Similarly, in patients with severe acute respiratory distress syndrome (ARDS) secondary to sepsis, intravenous uPA administration significantly improved arterial oxygenation without affecting bleeding or coagulation parameters [93].

### Combining profibrinolytic and anticoagulant intervention

The potential benefit of dual pro-fibrinolytic and anticoagulant therapies to prevent organ failure has been explored in animal models of DIC. For example, in an endotoxin-induced rabbit model of DIC, the combination of recombinant human tPA and r-hirudin reduced renal fibrin deposition. In a porcine model of E. coli–induced DIC, the combination of recombinant tPA and antithrombin significantly improved survival, hematocrit, antithrombin levels, and mitigated both macro- and microvascular organ damage [94]. In line with these findings, clinical studies have shown that recombinant human tPA improved organ perfusion in patients with fulminant meningococcemia [95,96], although at the cost of increased bleeding risk [97]. Importantly, data on the use of tPA in human sepsis-associated DIC remain limited. Given the dual risk of microthrombosis and bleeding in DIC, close clinical monitoring is essential to guide the safe application of these dual-targeted pharmacological strategies.

### Targeting the abnormal fragmentation of plasminogen by an elastase inhibitor

A recent study showed that DNA-bound elastase contributes to fibrinolytic insufficiency by degrading plasminogen, reducing its levels during excessive NETosis in sepsis-induced DIC [64]. Under normal conditions, plasminogen concentrations are not rate-limiting for plasmin formation; however, in sepsis, plasminogen levels eventually drastically reduced would impair the efficiency of its tPA-mediated activation. Restoring plasminogen to levels sufficient for full plasmin generation thus emerges as a critical step toward reestablishing effective fibrinolysis. Importantly, the pharmacological modulation of other actors in the fibrinolytic response, such as PAI-1 and tPA, in clinical trials or preclinical studies, has proven ineffective, most probably because their variations are not causative but rather coincidental to fibrinolytic insufficiency.

According to our observations [64], neutrophil elastase appears to be a significant contributor to the progression of sepsis-induced DIC. Sivelestat, a specific neutrophil elastase inhibitor, authorized in Japan for ARDS, has shown clinical benefits, including improved oxygenation, reduced need for mechanical ventilation, and shorter hospital stays [98]. A meta-analysis including 2,050 patients with acute lung injury or ARDS indicated that Sivelestat improved survival, enhanced oxygenation, and significantly reduced ICU stays in 1,069 patients [99].

In rats with septic shock induced by cecal ligation and perforation, studies further demonstrated that sivelestat improved survival, restored mean arterial pressure and glomerular filtration rate. It also reduced macrophage infiltration and the secretion of pro-inflammatory mediators [100]. Interestingly, a retrospective analysis involving septic patients with ARDS and DIC indicated that sivelestat treatment correlated with a lower lung injury score, an improved PaO_2_/FiO_2_ ratio, and a reduced ICU length of stay [101].

Building on these findings, a phase IIb clinical trial (NCT07214103) is now being launched to evaluate the efficacy of sivelestat in restoring fibrinolysis and improving clinical outcomes in patients with sepsis-induced DIC.

## Conclusion

Sepsis-induced DIC is characterized by a disrupted balance between coagulation and fibrinolysis, where fibrinolytic insufficiency plays a key role in sustaining microvascular thrombosis and organ dysfunction. While the underlying mechanisms—such as NET-mediated plasminogen degradation and excessive PAI-1 activity—are increasingly understood, translating these insights into effective therapies remains a challenge. Promising strategies targeting fibrinolytic insufficiency, including plasminogen restoration or combined anticoagulant–profibrinolytic approaches, are emerging but require validation in well-designed clinical trials. Ultimately, progress will depend on the development of reliable point-of-care tools to assess fibrinolytic status and guide personalized treatment. A better understanding of patient-specific profiles will be essential to improve outcomes in this complex and life-threatening syndrome.

## CRediT authorship contribution statement

All the authors have contributed to writing and have revised the manuscript.

## Consent for publication

NA.

## Ethics approval and consent to participate

NA.

## Funding

NA.

## Availability of data and materials

NA.

## Declaration of competing interest

JH has received honoraria for lectures from Pfizer PFE France, Sanofi Aventis France, Inotrem, MSD, Octapharma and Shionogi and is part of the steering committee from Bayer and AngioDynamics. The other authors have no conflict of interest to declare.
